# Vagus nerve stimulation allows to cease maintenance electroconvulsive therapy in treatment-resistant depression: a retrospective monocentric case series

**DOI:** 10.3389/fpsyt.2023.1305603

**Published:** 2024-01-30

**Authors:** Oumaima Aboubakr, Philippe Domenech, Isabelle Heurtebise, Raphaël Gaillard, Aurore Guy-Rubin, Romain Carron, Philibert Duriez, Philip Gorwood, Fabien Vinckier, Johan Pallud, Marc Zanello

**Affiliations:** ^1^Department of Neurosurgery, GHU Paris Psychiatrie et Neurosciences, Site Sainte-Anne, Paris, France; ^2^Université Paris Cité, Institute of Psychiatry and Neuroscience of Paris (IPNP), INSERM U1266, Paris, France; ^3^Department of Psychiatry, Service Hospitalo-Universitaire, GHU Paris Psychiatrie et Neurosciences, Site Sainte-Anne, Paris, France; ^4^Institut du Cerveau, Inserm U1127, CNRS UMR7225 Sorbonne Université, Paris, France; ^5^Cardiology Department Centre Hospitalier de Bourges, Bourges, France; ^6^Motivation, Brain, and Behavior (MBB) Lab, Paris Brain Institute (ICM) Hôpital Pitié-Salpêtrière, Paris, France; ^7^Clinique Villa Montsouris, Paris, France; ^8^Department of Functional and Stereotactic Neurosurgery, Timone University Hospital, Marseille, France; ^9^Aix Marseille Univ, APHM, INSERM, INS, Inst Neurosci Syst, Timone Hospital, Epileptology Department, Marseille, France; ^10^CMME Psychiatry Department, GHU PARIS Sainte-Anne, Paris, France; ^11^Laboratoire de Physiopathologie des Maladies Psychiatriques, Institute of Psychiatry and Neuroscience of Paris INSERM, Paris, France

**Keywords:** drug resistance, electric stimulation therapy, treatment outcome, safety, perception

## Abstract

**Context:**

The use of vagus nerve stimulation (VNS) to reduce or stop electroconvulsive therapy (ECT) in treatment-resistant depression seems promising. The aim of this study was to investigate the efficacy of VNS on the reduction of ECT sessions and mood stabilization.

**Methods:**

We conducted a monocentric retrospective case series of patients who suffered from treatment-resistant depression, treated with ECT and referred to our center for VNS. We investigated the number and the frequency of ECT sessions before and after VNS implantation. Secondary criteria consisted in the Montgomery Åsberg Depression Rating Scale (MADRS) score, number of medical treatments, dosage of the main treatment and length of hospital stays before and after VNS. Additionally, we sent an anonymous survey to psychiatrists and other physicians in our institution to investigate their knowledge and perception of VNS therapy to treat treatment-resistant depression.

**Results:**

Seven patients benefited from VNS: six (86%) were female (mean age of 51.7 +/− 16.0 years at surgery), and five (71%) suffered from bipolar depression (three type I and two type II). All patients were followed up at least 2 years post-implantation (range: 27–68 months). Prior to VNS, six patients were treated by maintenance ECT. After VNS, three (43%) patients did not require maintenance ECT anymore, and three (43%) patients required less frequent ECT session with a mean 14.7 +/− 9.8 weeks between sessions after VNS vs. 2.9 +/− 0.8 weeks before VNS. At last follow-up, 4 (57%) patients had stopped ECT. Five (71%) patients implanted with VNS were good responders (50% decrease relative to baseline MADRS). According to the survey, psychiatrists had a significantly better perception and knowledge of ECT, but a worse perception and knowledge of VNS compared to other physicians.

**Conclusion:**

VNS is a good option for treatment-resistant depression requiring maintenance ECT dependence. Larger on-going studies will help broaden the implanted patients while strengthening psychiatrists’ knowledge on this therapy.

## Introduction

According to the World Health Organization (WHO), over 300 million people are estimated to suffer from depression, equivalent to 4.4% of the world’s population (1). Approximately 30% of depressive patients are treatment-resistant (2, 3). Electroconvulsive therapy (ECT) is the standard treatment for treatment-resistant depression (4). It is recognized as efficient for mood stabilization but is associated with several issues, such as its long-term side effects (headaches, memory loss), a poor acceptability, and a high rate of relapse after ECT interruption (5–8). The necessity for maintenance ECT is challenging in terms of hospital resources and costs. More recently, Abrupt discontinuation of maintenance ECT during COVID-19 pandemic lead to relapses and highlighted the need for alternative therapy (9–11).

Vagus Nerve Stimulation (VNS) has been approved by the FDA as a treatment option for treatment-resistant depression since 2005 in the US and long-term follow-up of large cohorts revealed its efficacy in treatment-resistant depression (12). It is possible to perform ECT while having a VNS device and a previous case series described VNS as a potential relay to progressively cease maintenance ECT (13).

In France, VNS is still not recommended for treatment-resistant depression: it remains only offered to a few patients in tertiary care centers based on humanitarian exemptions. The referral of potential candidates to VNS remains a challenge, which makes VNS hardly accessible to most patient suffering treatment resistant depression (14). The main goal of this study was to assess the efficacy of VNS on maintenance ECT weaning and on depressive mood stabilization in treatment-resistant depression. The GHU PARIS Hospital (Paris, France) was born after the merger of the Sainte Anne Hospital, the Maison Blanche Hospital, and the Perray-Vaucluse Hospital in 2019. Due to its large coverage of the Ile de France region (representing approximatively 20% of the French population), GHU PARIS Hospitals takes care of approximatively 1 people on 40 in that region. If there is a large majority of psychiatrists, the GHU PARIS hospital medical population also includes general care practitioners, intensive care specialists, neurologists, neuroradiologists, specialists of physical and functional rehabilitation, and neurosurgeons with a tradition of multidisciplinary dialogue (15).

The main objective of the study was to retrospectively collected data concerning efficacy and safety of VNS for treatment-resistant depression after maintenance ECT. The second objective was to review psychiatrists and non-psychiatrists’ knowledge and perception of ECT and VNS as treatment options for depression using an anonymous online survey, in order to understand the low number of patients referred to VNS surgery after maintenance ECT.

## Methods

### Study design – settings and timeframes

This study is a retrospective, monocentric case series (tertiary care center, GHU PARIS Hospital, France). One investigator (O.A) collected clinical, imaging, surgical, treatment-related and follow-up data for all patients who underwent VNS surgery for treatment-resistant depression using a protocol designed for this study. This case series has been reported in line with the Preferred Reporting Of CasE Series in Surgery (PROCESS) Guidelines (16). The period of interest was from January 2015 to January 2020. Post January 2020, the COVID pandemic stopped these compassionate surgeries. The GHU PARIS Hospital (France) is a tertiary care center with a dedicated functional neurosurgery team and a dedicated psychiatry team.

### Participants – registration

Inclusion criteria were: (1) patients older than 18 at surgery; (2) treatment resistant depression (unipolar or bipolar); (3) implantation with a VNS system; (4) available data. Exclusion criteria were: (1) patients lost to follow-up (no contact with the medical team from GHU PARIS Sainte Anne during the last year); (2) follow-up shorter than 2 years.

The collected data included patient demographics (sex, profession, age at diagnosis, personal and family medical history), clinical characteristics (symptoms at diagnosis, number and severity of episodes, hospital stays, suicide attempts), imaging data when available, medical treatment details in particular dosage of main therapy, ECT details, surgical and post operative data.

All patients filled a signed informed consent concerning the use of their de-identified data for scientific purpose. The study was conducted in accordance with the Declaration of Helsinki. The local institutional review board approved the study protocol (IRB00011687).

### Intervention

Patients who were referred by their psychiatry team to a functional neurosurgeon for a neuromodulation treatment option were assessed and implanted with a VNS device (Demi-Pulse^®^, LivaNova, United States) on the left side. The surgical technique was previously described (17). Briefly, the patients were under general anesthesia on supine position, the vagus nerve dissection and placing the helical coils around the nerve were performed under optical magnification. Stimulation was activated between 1- to 16 weeks after the operation at the standard parameters used for treatment resistant epilepsy. The intensity of stimulation was gradually increased to maximize its efficacy while minimizing sides effects.

### Follow-up and efficacy assessment

Follow-up was conducted jointly by the psychiatry and the neurosurgery team through clinical consultations. Patients were followed between 2 and 5 years post-operatively with repeated measurements of the MontgomeryÅsberg Depression Rating Scale (MADRS). It is a ten-item diagnostic scale for depression, designed to be sensitive to treatment effect, validated in several languages including French and widely used (18, 19).

The interruption or reduction of ECT sessions after VNS activation was the primary outcome. Secondary outcomes were: 2/difference between MADRS scores obtained in the month preceding VNS activation and at last follow-up; 3/the number of medications and changes in dosage of the main treatment in the month preceding VNS activation and at last follow-up; 4/length of hospitalization in a psychiatric Department before and since VNS activation (measured in days) until last follow-up.

### Survey

An anonymous survey was sent to psychiatrists and other physicians (general practitioners, neurologists, neurosurgeons, and intensive care specialists) working at GHU PARIS Hospital via Google Forms. This 13-items questionnaire was designed by a multidisciplinary team including 2 senior neurosurgeons, and 3 senior psychiatrists (see Supplementary Table S1). A paired Likert score ensured proper comparability between answers. A scale ranging from 1 to 4 was used, with 1 corresponding to “Very good,” and 4 “Bad.” There was no neutral proposition (forced answers). The questionnaire included: 5 items concerning individual participants and local organization (specialization of the participants, awareness of the multidisciplinary meeting, etc.), 8 items concerning the neuromodulation procedure (knowledge and perception) dealing with ECT, VNS but also repetitive trans magnetic stimulation (rTMS) and deep brain stimulation (DBS). A free comment section was provided at the end of the questionnaire. Answers were binarized into positive answers for 1 & 2 (“very good” and “good,” respectively) and negative answers for 3 & 4 (“mediocre” and “bad,” respectively).

The questionnaire was sent by e-mail to 587 physicians working at GHU PARIS Hospital. Reminder e-mails were sent 2 weeks and 4 weeks after the initial email.

### Measurements and analysis

Categorical variables were described as number and percentages. Continuous variables were described as mean ± standard deviation. Univariate analyses were carried out using the chi-square test after converting Likert’s scale data into binary variables when required. A value of *p* of less than 0.05 was considered significant. Analyses were performed using Jamovi (20).

## Results

### Patients’ characteristics

Table 1 summarizes patients’ characteristics.

**Table 1:** Patients’ characteristics.

	Patient 1	Patient 2	Patient 3	Patient 4	Patient 5	Patient 6	Patient 7
Gender	F	F	F	F	F	F	M
Age (years)	43	22	53	74	58	54	58
Diagnosis	Bipolar disorder, type I	Bipolar disorder, type II	Depression disorder	Depression disorder	Bipolar disorder, type II	Bipolar disorder, type I	Bipolar disorder, type I
Comorbidities	Generalized Anxiety Disorder	Epilepsy	Anorexia		Anorexia Substance abuse disorder		
Clinical course before VNS (years)	14	9	5		12	22	23
Number of medications	>10	>10	>10	3	4	>10	2
Clozapine	Yes	No	No	No	No	Yes	No
ECT	Yes	Yes	Yes	Yes	Yes	Yes	Yes
rTMS	Yes	No	No	No	No	Yes	No
Ketamine perfusions	Yes	Yes	Yes	No	No	No	No
Time since VNS intervention (years)	2	3	4	4	2	2	4
VNS activation status	On	On	On	On	On	Off	On
Short term complications	No	No	No	No	No	No	Yes (dysphonia)
Long term complications	Yes	No	No	No	Yes	No	No
Second surgery	Yes				No		No

ECT, electroconvulsive therapy; rTMS, repetitive transcranial magnetic stimulation; VNS, Vagus nerve stimulation.

Since March 2017, seven patients were implanted with VNS for treatment-resistant depression (five bipolar and two unipolar) at GHU PARIS Hospital’s Neurosurgery department. Patients’ characteristics are detailed in Table 1. Six patients were female, the mean age at implantation was 51 years (range 22–74). Three patients were also diagnosed with other psychiatric conditions (anorexia, generalized anxiety disorder and substance abuse disorder). Five patients have a close family history of psychiatric disorder (mood disorders, substance abuse disorder, suicide). Four patients have attempted suicide at least once. One patient happens to also have epilepsy (VNS surgery for treatment-resistant depression only).

The median delay to surgery was equal to 13 years (range 5–23 years) between diagnosis and referral for VNS. At surgery, all patients had received several medical treatments consisting in antidepressants, mood regulators and neuroleptics (four out of seven had received more than 10 different drugs). Two patients had received a treatment by clozapine and three patients had tried ketamine intravenous perfusions. As for non-pharmaceutical treatments, all patients had received ECT, and two patients had also received repetitive rTMS. Six patients were on maintenance ECT at the time of surgery.

All patients were followed up at least 2 years post-implantation (mean: 43.9 +/− 14.3 months, range: 27–68 months). After VNS implantation, one patient experienced a short-term complication (transitory voice alteration) and two patients experienced long term complications (Supplementary Figures S3, S4; Supplementary Video 1): sternocleidomastoid muscle contraction likely caused by the involuntary stimulation of the superior root of the ansa cervicalis (21), and severe sinus bradycardia, a rare complication of VNS (22–25), respectively. Muscle contraction disappeared after a revision surgery with lead replacement for the first patient whereas the implantation of a pacemaker allowed to restart VNS for the second one. The median activation period was 36 months (range 12–64). At last follow-up, six VNS devices were still activated. The only deactivated stimulator was deactivated at the patient’s request (chest discomfort without dyspnea).

### Efficacy of VNS on decreasing the use of ECT

**Figure 1:**
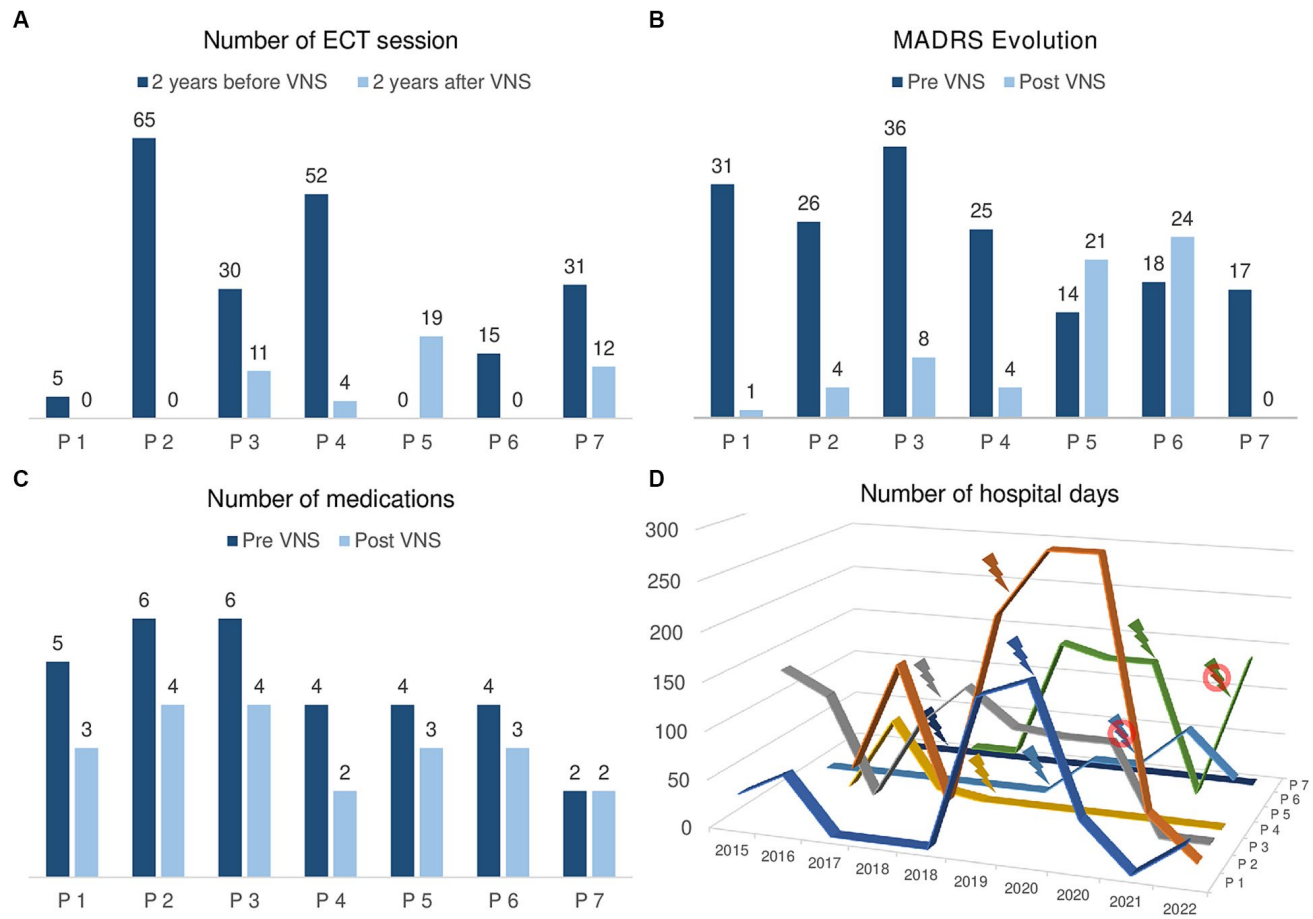
Efficacy of VNS on mood stabilization in the case of 7 patients stimulated at GHU PARIS Hospital. **(A)** Number of ECT sessions in the 2 years before VNS activation and in the 2 years after VNS activation. Patients 1, 2, 3, 4, 6, and 7 required less ECT sessions in the 2 years after VNS activation. Only patient 5 received 19 sessions in the 2 years after VNS versus 0 in the 2 years before. **(B)** MADRS score before VNS activation and at last follow-up. Patients 1, 2, 3, 4, and 7 show a reduction of their MADRS score and are in remission (MADRS ≤9). Clinically, patients 1, 2, 4, and 7 are in remission and patient 3 is experiencing a mild depressive episode. Patients 5 and 6 show a higher MADRS score at last follow-up than before VNS. Clinically, they are hospitalized in a psychiatry ward for a recurrent depressive episode. **(C)** Number of medications prescribed before VNS activation and at last follow-up. Patient 7 has been consistently prescribed 2 medications and all the other patients take less medications at last follow-up. **(D)** Number of days per year spent in a psychiatric ward in the 2 years before and after VNS activation. Of note, patient 7 has never been admitted to psychiatry. Patients 1 to 4 show a tendency towards less hospitalizations since VNS activation. Patients 5 and 6 are currently hospitalized in a psychiatry ward.

Figure 1 presents the results of VNS on several efficacy criteria.

Since all patients received ECT before being referred to neurosurgery for VNS, we documented the number of sessions they received in the 2 years before VNS and in the 2 years following VNS activation (Figure 1A).

Three (43%) patients did not require any ECT in the 2 years following VNS activation. Three (43%) patients could reduce ECT frequency in the 2 years following VNS activation with a mean 14.9 +/− 9.8 weeks between ECT sessions vs. 2.9 +/− 0.8 weeks in the 2 years before VNS. Only one patient received 19 ECT sessions in the 2 years following VNS activation vs. 0 in the 2 years before VNS: it was the patient suffering from the severe sinus bradycardia with a deactivated VNS. At last follow-up, 4 (67%) patients had stopped ECT and the patient requiring a pacemaker implantation showed a favorable evolution after VNS activation. No adverse effect occurred during ECT sessions after VNS implantation.

### Efficacy of VNS on mood stabilization

Regarding VNS efficacy based on MADRS score, five patients showed a positive response with a reduction of their MADRS score (Figure 1B). Four patients (1, 2, 4, and 7) are currently in clinical remission (MADRS ≤4), euthymic and living at home. Patient 3 is receiving outpatient intravenous ketamine perfusions for a mild recurrent depressive episode (MADRS = 8 vs. 36 before VNS). Patients 5 and 6 are hospitalized in a psychiatry Department for a recurrent depressive episode.

We observed a reduction of the total number of medications prescribed for all but one patient who has been consistently prescribed 2 medications (Theralite and Carbamazepine) before and after VNS activation (Figure 1C). The mean reduction was of 1.4 +/− 0.8 treatment with a decrease in dosage of the main treatment of 38.3% +/− 35.1 (4 patients took Lithium, 2 anti-psychotic medications, and 1 a dopamine agonist).

There was a general trend towards less hospitalized days in a psychiatric department after the VNS activation in comparison with the baseline period (Figure 1D), but with important individual variations: for instance, patient 1 spent 36 days hospitalized after VNS surgery vs. 176 before whereas patient 3 was hospitalized 135 days after VNS surgery vs. 136 before.

There were no suicide following VNS activation and one episode of self-harm in a patient suffering from numerous self-harm episodes prior to VNS activation.

### GHU PARIS medical population survey: psychiatrists and other physicians’ knowledge and perception of ECT and VNS

**Figure 2:**
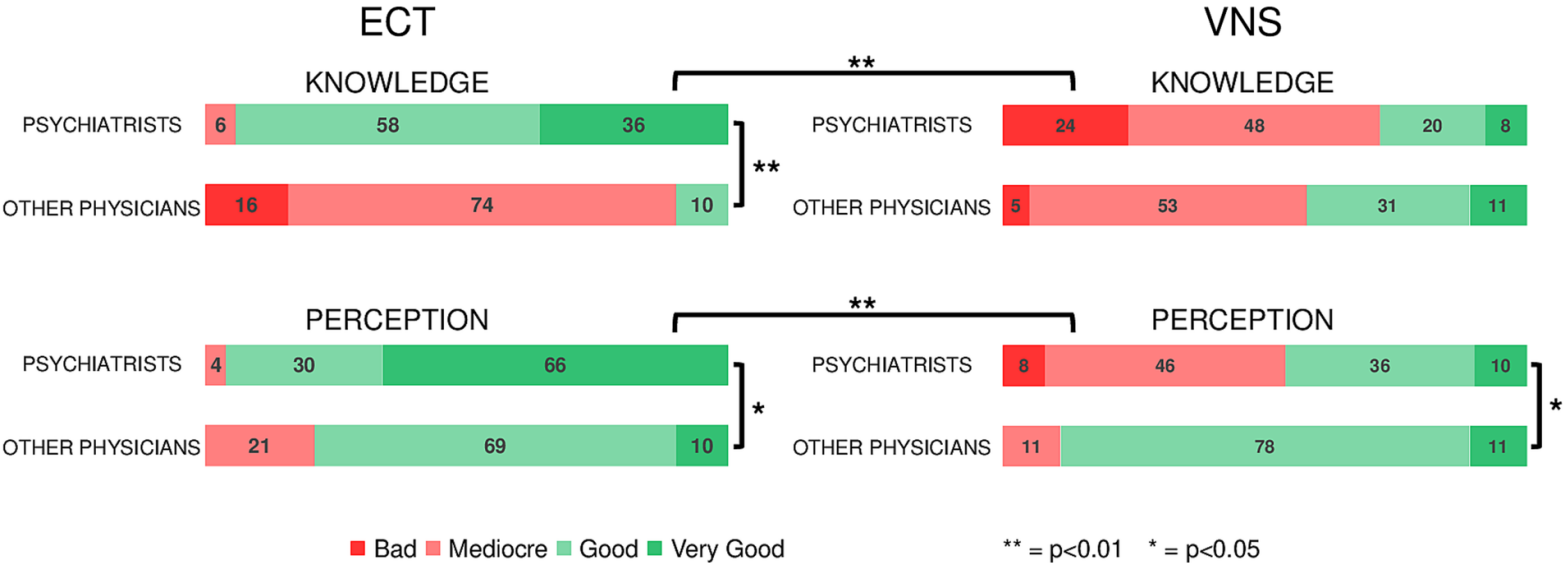
Results of the anonymous survey sent to psychiatrists and other physicians working at GHU PARIS Hospital, assessing their knowledge and perception of neuromodulation treatments for depression (ECT and VNS). 94% of psychiatrists vs. 10% of other physicians report a good (very good + good) knowledge of ECT (*p* < 0.001) and 96% of psychiatrists have a good perception of ECT vs. 79% of physicians (*p* = 0.027). 28% of psychiatrists vs. 42% of physicians report a good knowledge of VNS (*p* = 0.268) whereas 46% of psychiatrists vs. 89% of physicians have a good perception of VNS (*p* = 0.007). Psychiatrists have a significantly worse knowledge of VNS compared to ECT (*p* < 0.001). Their perception of VNS was the worse among the four investigated neuromodulation techniques (*p* < 0.001 vs. ECT).

Figure 2 summarize the results of survey analysis.

Response rate to the survey was 13.5% 50 psychiatrists and 19 other physicians (2 general practitioners, 2 intensive care specialists, 7 neurologists, and 8 neurosurgeons).

Regarding ECT, 94% of psychiatrists vs. 10% of other physicians reported a good (very good + good) knowledge of the procedure (*p* < 0.001) and 96% of psychiatrists had a good perception of ECT vs. 79% of other physicians (*p* = 0.027). By contrast, 72% of psychiatrists vs. 58% of other physicians reported a bad (mediocre + bad) knowledge of VNS and 54% of psychiatrists had a bad perception of VNS vs. 11% of other physicians (*p* < 0.001). Psychiatrists had a significantly poorer knowledge of VNS compared to ECT (*p* < 0.001). Their perception of VNS was the worse among the four investigated neuromodulation techniques (*p* < 0.001 vs. ECT). The results for deep brain stimulation (DBS) and repetitive trans magnetic (rTMS) are reported in Supplementary Figure S5.

## Discussion

### Key results

This study showed that: 1/VNS could contribute to cease or reduce the frequency of maintenance ECT, 2/after VNS, the majority of patients had fewer medications and/or fewer recurrences and/or shorter hospital stays, 3/VNS in treatment-resistant depression, unipolar or bipolar, was successful in mood stabilization according to MADRS, 4/psychiatrists at a tertiary care center had a poor knowledge and perception of VNS and in general of invasive neuromodulation therapies.

### Interpretation

About 50% of patients with major depression relapse within 1 year of treatment with ECT but maintenance ECT remains discussed, due to neurocognitive adverse effects of ECT (26, 27). During COVID-19 pandemic, nearly 60% of the patients requiring maintenance ECT relapsed after abrupt discontinuation (9–11). It has been reported that VNS can help to decrease frequency or to stop maintenance ECT (13, 28, 29). Our results were in line with these results: all the patients with the VNS activated at least 2 years after the implantation performed less ECT session than before VNS implantation and 4 out 5 totally stopped maintenance ECT. Moreover, maintenance ECT has a significant cost: reducing the frequency of ECT session at the cost of a VNS implantation is economically sound (28). As previously described, none complication occurred during ECT session after VNS implantation: it is another argument to propose VNS in front of an ECT dependence (29, 30).

The link between maintenance ECT and VNS is not evident. Mechanisms of action of both techniques are not fully understood (31, 32). Some directions could be: the role of the neuro-endocrine system as ECT and VNS both exert an effect on it (31, 33); the need to disturb causal depression network as VNS is known to perturb epileptic aberrant network (34, 35); the effect of neurogenesis with an increase in hippocampal volume after VNS or after ECT (36, 37). It is probably the conjunction of several mechanisms of action that explained the therapeutic effect of both techniques.

This cases series was another step towards the confirmation of VNS efficacy for treatment resistant depression: five patients had favorable outcomes after VNS activation despite being considered after the failure of more than 4 different medications and the bad tolerance, non-response, exhaustion, reliance on ECT treatment. Apart from MADRS score, length of hospitalization, number of medication and number of ECT sessions were globally reduced. This is in line with other studies and should be confirmed by larger studies (12, 38–42). There were no suicide following VNS activation and one episode of self-harm in a patient suffering from numerous self-harm episodes prior to VNS activation. The other complications rate was higher compared to previous literature, probably due to the small sample size (12). It should be stressed that the VNS efficacy and tolerance was correct in a population mainly made up of patients suffering from bipolar disorder, making VNS a potential treatment of choice for this subpopulation (12).

The paucity of patients suffering from treatment-resistant depression referred to VNS surgery was in line with previous results (43, 44). Beside the difficult definition of treatment-resistance in psychiatry, several reasons could be provided: the psychiatrists’ residency offers only limited contact with neuromodulation, only few hospitals have enough resources to take care of treatment-resistant psychiatric patients, perception of medical invasiveness is highly variable, psychiatrists have little knowledge on current neurosurgical procedures, and literature is not straightforward (45–47). The anonymous survey provided additional evidence that psychiatrists working at a tertiary care center did not have enough knowledge on invasive neuromodulation such as VNS whereas ECT was well-known. There was a significant difference between psychiatrists and other physicians in term of invasive neuromodulation perception, even if their knowledge was not significantly different. There is a need for better teaching of psychiatric neurosurgery for both residents and seniors physicians (46, 48, 49).

### Limitations

These findings should be interpreted with caution, given the retrospective and monocentric design, the lack of a control group, all limiting the generalizability of the results. The specific medical population and the low response rate weaken the survey analysis. Further confirmatory analyses are required to reproduce the present results.

## Conclusion

This case series adds to the growing literature concerning VNS usefulness in case of maintenance ECT. VNS did not preclude to perform ECT sessions after the implantation but help to reduce the frequency or even to stop maintenance ECT. Large ongoing studies, such as the RECOVER study, on VNS in treatment-resistant depression will help to precise the appropriate place of VNS in the treatment algorithm for treatment-resistant depression and will ease the referral of patients to surgery (50).

## Data availability statement

The raw data supporting the conclusions of this article will be made available by the authors, without undue reservation.

## Ethics statement

The studies involving humans were approved by Campus de Neurochirurgie – IRB00011687. The studies were conducted in accordance with the local legislation and institutional requirements. Written informed consent for participation was not required from the participants or the participants’ legal guardians/next of kin in accordance with the national legislation and institutional requirements.

## Author contributions

OA: Data curation, Investigation, Writing – original draft, Writing – review & editing. PDo: Data curation, Investigation, Validation, Writing – original draft, Writing – review & editing. IH: Data curation, Investigation, Writing – original draft, Writing – review & editing. RG: Conceptualization, Data curation, Formal analysis, Writing – original draft, Writing – review & editing. AG-R: Formal analysis, Investigation, Writing – original draft, Writing – review & editing. RC: Data curation, Formal analysis, Writing – original draft, Writing – review & editing. PDu: Methodology, Writing – original draft, Writing – review & editing. PG: Formal analysis, Writing – original draft, Writing – review & editing. FV: Investigation, Writing – original draft, Writing – review & editing. JP: Data curation, Formal analysis, Investigation, Methodology, Project administration, Writing – original draft, Writing – review & editing. MZ: Conceptualization, Data curation, Formal analysis, Investigation, Methodology, Supervision, Validation, Writing – original draft, Writing – review & editing.
