# Supplementary material for: Vagus nerve stimulation allows to cease maintenance electroconvulsive therapy in treatment-resistant depression: a retrospective monocentric case series
Source: Front Psychiatry. 2024 Jan 30;14:1305603. doi: 10.3389/fpsyt.2023.1305603 (PMC10861730; doi:10.3389/fpsyt.2023.1305603)
Supplement: Supplementary file 1 [file Table_1.docx]

Supplementary Table 1. Form used for the survey.

| **1** | **What is your specialty ?** | | | | | | | |
| --- | --- | --- | --- | --- | --- | --- | --- | --- |
|  | Psychiatry | Neurology | | Neurosurgery | | General Practitioner | | Intensivist |
| **2** | **What is your knowledge of ECT ? (indications, process, potential complications, follow-up)** | | | | | | | |
|  | Very good | | Good | | Mediocre | | Bad | |
| **3** | **What is your perception of ECT (risk-benefit ratio, indications) ?** | | | | | | | |
|  | Very good | | Good | | Mediocre | | Bad | |
| **4** | **What is your knowledge of rTMS ? (indications, process, potential complications, follow-up)** | | | | | | | |
|  | Very good | | Good | | Mediocre | | Bad | |
| **5** | **What is your perception of rTMS (risk-benefit ratio, indications) ?** | | | | | | | |
|  | Very good | | Good | | Mediocre | | Bad | |
| **6** | **What is your knowledge of VNS ? (indications, process, potential complications, follow-up)** | | | | | | | |
|  | Very good | | Good | | Mediocre | | Bad | |
| **7** | **What is your perception of VNS (risk-benefit ratio, indications) ?** | | | | | | | |
|  | Very good | | Good | | Mediocre | | Bad | |
| **8** | **What is your knowledge of DBS ? (indications, process, potential complications, follow-up)** | | | | | | | |
|  | Very good | | Good | | Mediocre | | Bad | |
| **9** | **What is your perception of DBS (risk-benefit ratio, indications) ?** | | | | | | | |
|  | Very good | | Good | | Mediocre | | Bad | |
| **10** | **Are you aware of the bimonthly neuromodulation multidisciplinary meeting about invasive techniques (VNS, DBS) involving psychiatrists, neurologists, neuroradiologists, anesthesiologists, intensivists, and neurosurgeons at GHU PARIS Hospital ?** | | | | | | | |
|  | Yes | | | | No | | | |
| **11** | **Are you informed on the options of care offered by the Federation of Neurostimulation regarding noninvasive neuromodulation ? (ECT, rTMS, tDCS)?** | | | | | | | |
|  | Yes | | | | No | | | |
| **12** | **Would you be interested in discussing a patient’ case during a neuromodulation multidisciplinary meeting ?** | | | | | | | |
|  | Yes | | | | No | | | |
| **13** | **Would you be interested in a Cloud for physicians to share information on neuromodulation ?** | | | | | | | |
|  | Yes | | | | No | | | |
| **Free comments** | | | | | | | | |

ECT: Electroconvulsive Therapy, rTMS: repetitive Trans Magnetic Stimulation, VNS: Vagus Nerve Stimulation, DBS: Deep Brain Stimulation
