# Supplementary figures and images for: Vagus nerve stimulation allows to cease maintenance electroconvulsive therapy in treatment-resistant depression: a retrospective monocentric case series
Source: Front Psychiatry. 2024 Jan 30;14:1305603. doi: 10.3389/fpsyt.2023.1305603 (PMC10861730; doi:10.3389/fpsyt.2023.1305603)

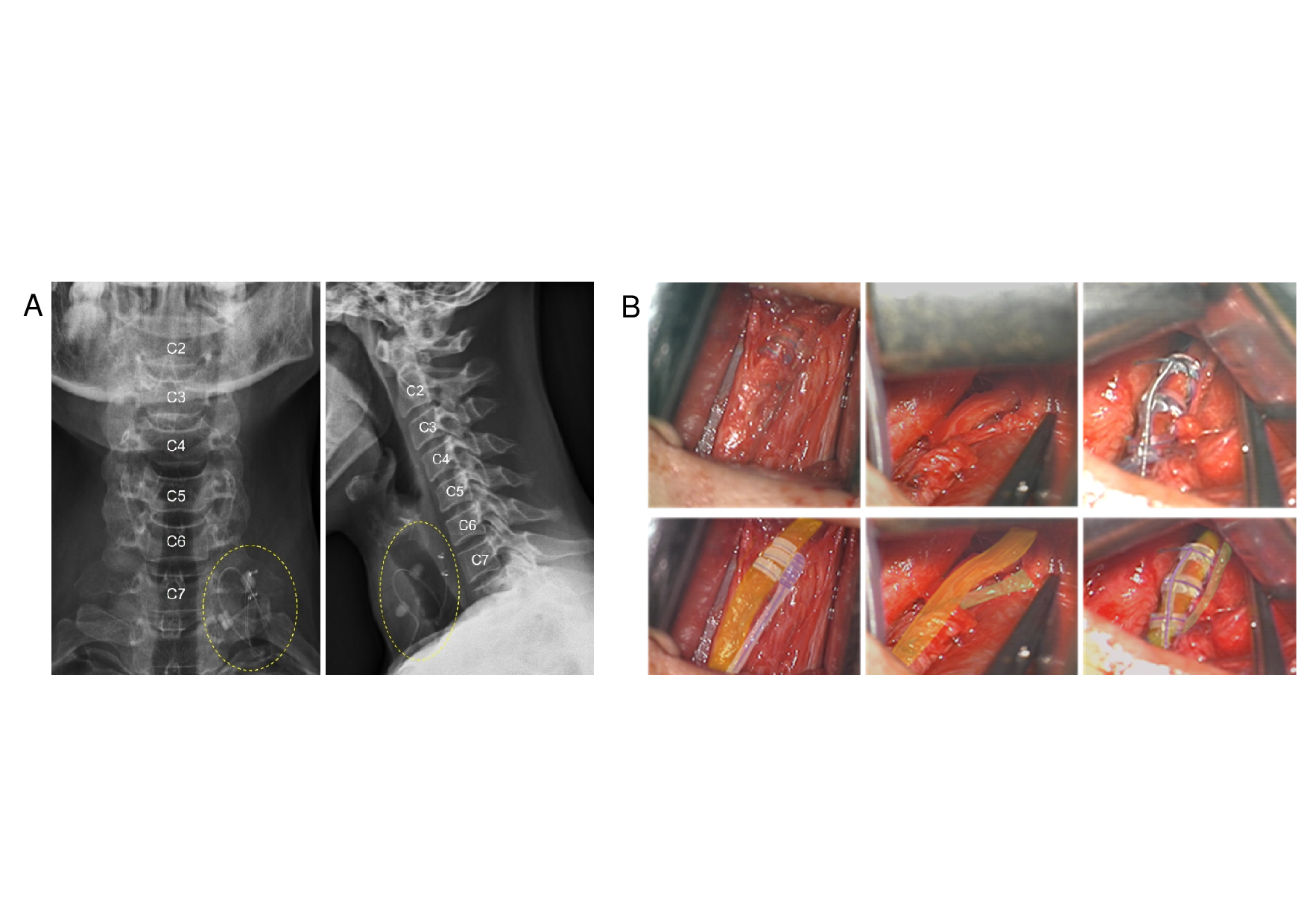

Supplement: Supplementary file 3 [file Image_1.TIF]

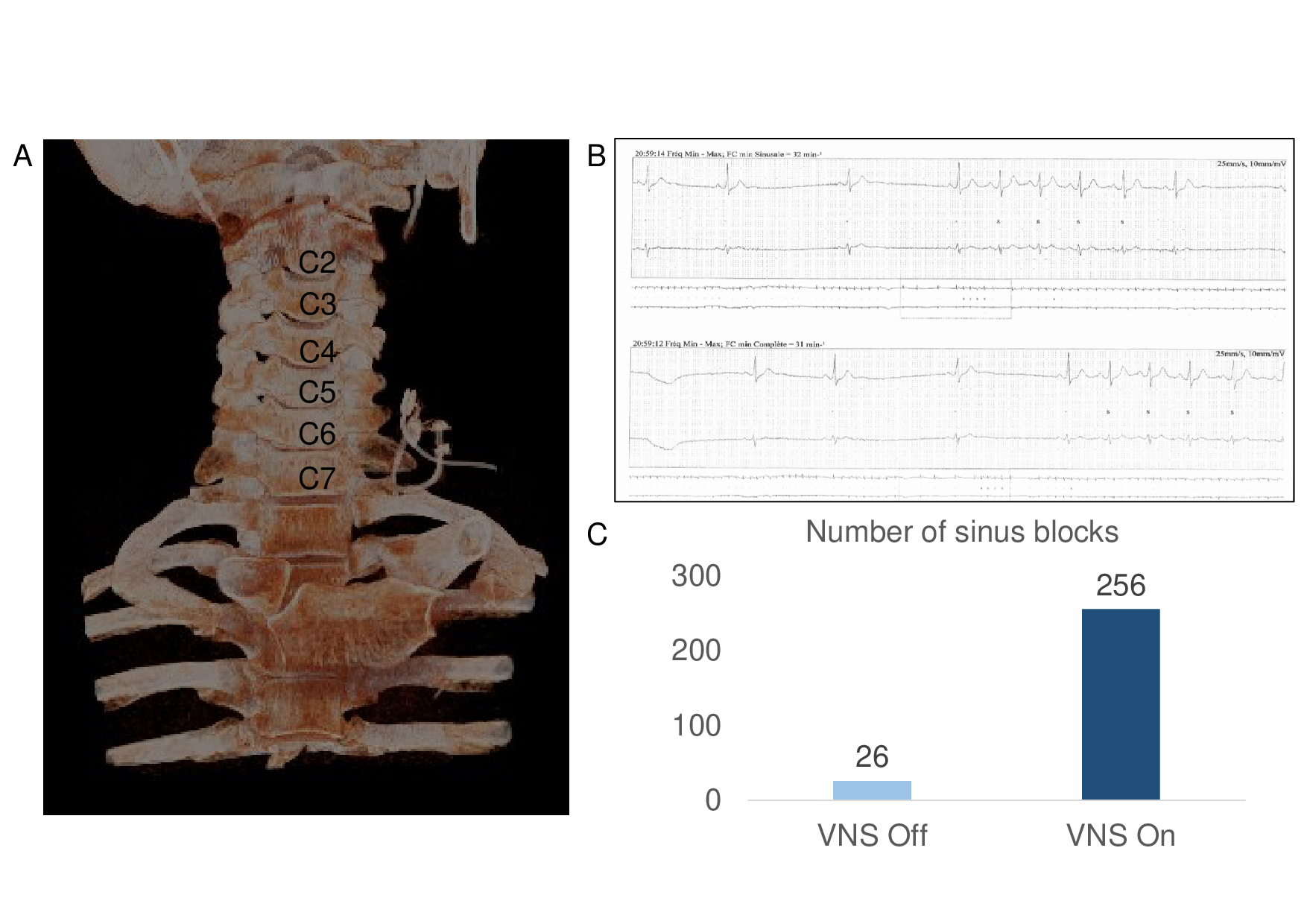

Supplement: Supplementary file 4 [file Image_2.TIF]

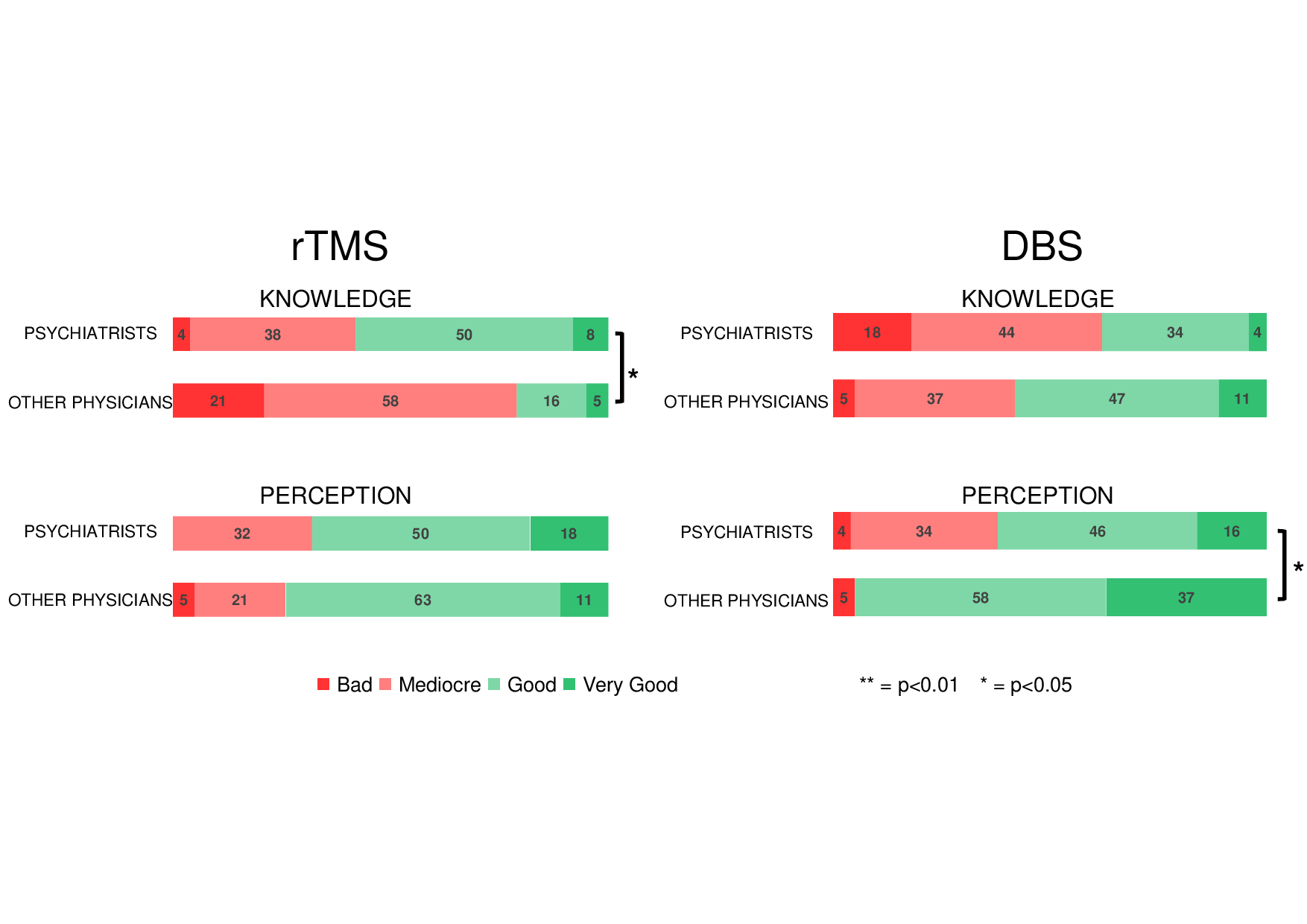

Supplement: Supplementary file 5 [file Image_3.TIF]
